# Spatial variability of forward modelled attenuated backscatter in clear‐sky conditions over a megacity: Implications for observation network design

**DOI:** 10.1002/qj.4253

**Published:** 2022-03-10

**Authors:** Elliott Warren, Cristina Charlton‐Perez, Humphrey Lean, Simone Kotthaus, Sue Grimmond

**Affiliations:** ^1^ Department of Meteorology University of Reading Reading UK; ^2^ Met Office Exeter UK; ^3^ Met Office@Reading Reading UK; ^4^ Institut Pierre Simon Laplace (IPSL), CNRS, École Polytechnique, Institut Polytechnique de Paris Palaiseau Cedex France

**Keywords:** aerosols, lidar, network design, numerical weather prediction, urban

## Abstract

Sensors that measure the attenuated backscatter coefficient (e.g., automatic lidars and ceilometers [ALCs]) provide information on aerosols that can impact urban climate and human health. To design an observational network of ALC sensors for supporting data assimilation and to improve prediction of urban weather and air quality, a methodology is needed. In this study, spatio‐temporal patterns of aerosol‐attenuated backscatter coefficient are modelled using Met Office numerical weather prediction (NWP) models at two resolutions, 1.5 km (UKV) and 300 m (London Model [LM]), for 28 clear‐sky days and nights. Initially, attenuated backscatter coefficient data are analysed using S‐mode principal component analysis (PCA) with varimax rotation. Four to seven empirical orthogonal functions (EOFs) are produced for each model level, with common EOFs found across different heights (day and night) for both NWP models. EOFs relate strongly to orography, wind, and emissions source location, highlighting these as critical controls of attenuated backscatter coefficient spatial variability across the megacity. Urban–rural differences are largest when wind speeds are low and vertical boundary‐layer dynamics can more effectively distribute near‐surface aerosol emissions vertically. In several night‐time EOFs, gravity‐wave features are found for both NWP models. Increasing the horizontal resolution of native ancillaries (model input parameters) and improving the urban surface scheme in the LM may enhance the urban signal in the EOFs. PCA output, with agglomerative Ward cluster analysis (CA), minimises intra‐group variance. The UKV and LM CA shape and size results are similar and strongly related to orography. PCA‐CA is a simple, but adaptable methodology, allowing close alignment with observation network design goals. Here, CA is used with wind roses to suggest the optimised ALC deployment is one in the city to observe the urban plume and others surrounding the city, with priority given to cluster size and frequency of upwind advection.

## Graphical abstract

Principal component analysis and cluster analysis are used with forward‐modelled aerosol attenuated backscatter from two numerical weather prediction models over Greater London. This identified regions with common spatial patterns in backscatter variability, which can subsequently help inform lidar deployment in an urban network. Common patterns strongly relate to relative aerosol emission locations, winds, and orography, factors that should be considered in future instrument network deployments.

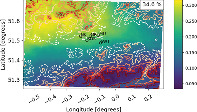

## 1 INTRODUCTION

Automatic lidars and ceilometers (ALCs) measure the attenuated backscatter coefficient from atmospheric particles. Although many were originally used to measure cloud base height (Illingworth *et al*., 2007), there are an increasingly wider variety of applications, including investigation of particle mass concentrations (Münkel and Roininen, 2010; You *et al*., 2016), to measure boundary‐layer height (Wang *et al*., 2012; Peña *et al*., 2013; Kotthaus and Grimmond, 2018b), and sea‐breeze dynamics (Lemonsu *et al*., 2006). As instrument sensitivity has increased (Kotthaus *et al*., 2016), the utility of ALC‐measured aerosol backscatter has grown (Illingworth *et al*., 2013; Madonna *et al*., 2014; Jin *et al*., 2018; Cimini *et al*., 2020), though many studies analyse relative variations or the signal‐to‐noise ratio of the measurements (Wiegner and Gasteiger, 2015). To relate modelled aerosol fields to the observed aerosol‐attenuated backscatter coefficient directly, a forward operator (FO) is required.

An aerosol FO estimates the attenuated backscatter coefficient using aerosol variables as inputs. Typically, model aerosol‐variable inputs are used from numerical weather prediction (NWP) or chemistry transport models to run an FO, so that the estimated attenuated backscatter coefficient can be evaluated against ALC observations or the latter can be assimilated (Benedetti and Dabas, 2016; Charlton‐Perez *et al*., 2016; Geisinger *et al*., 2017; Chan *et al*., 2018; Warren *et al*., 2018). Several of these studies take advantage of ALC networks to obtain spatial aerosol information.

ALC networks cover spatial scales spanning cities (Kotthaus and Grimmond, 2018a), countries (Flentje *et al*., 2010; Osborne *et al*., 2018), and continents (Welton *et al*., 2000; Illingworth *et al*., 2007, 2015; Pappalardo *et al*., 2014; Guerrero‐Rascado *et al*., 2016; Nishizawa *et al*., 2016). However, the ideal positioning of an ALC within a network is uncertain and may depend on the location and spatial scale of dominant features, whether local, meso, or synoptic scale. Undesirable observational redundancy can exist when multiple ALCs are positioned such that they are capturing the same meteorological features and respond to the same emission sources, while observations may miss (or only partially capture) features occurring elsewhere. Optimised instrument positioning maximises sampling efficiency and strengthens the observation products acquired across a network for a given equipment and operational cost.

Though practical constraints will impact final deployment locations (e.g., geopolitical boundaries or suitability of local infrastructure), an understanding of spatial variability and scale of meteorological features and their interaction with emission sources in the study area is critical. Most relevant processes are expressed in dynamics of the near‐surface atmosphere; that is, the atmospheric boundary layer (ABL).

Network design for weather and hydrological instrumentation has been informed by the analysis of spatial or spatio‐temporal variability in meteorological observations using (geo‐)statistical techniques (e.g., Bastin *et al*., 1982; Burn and Goulter, 1991; Bayat *et al*., 2019), and regional climate models' climatological variability (St‐Hilaire *et al*., 2003). However, the resolution of the latter can be coarse compared with many meso‐scale and local‐scale processes driving aerosol distributions, and higher resolution models might better represent smaller scale processes that could be important.

The principal component analysis (PCA) technique can reduce a dataset to a series of orthogonal functions, or modes, that represent patterns of variability (Wilks, 2011). S‐mode PCA (or empirical orthogonal function [EOF] analysis) is one variant that focuses on identifying spatial patterns in the different eigenvectors. It is used extensively to analyse meteorological variability, including rainfall (Smith and Phillips, 2013; Yu and Lin, 2015), wind (Álvarez‐García *et al*., 2020; Farjami and Hesari, 2020), and temperature (Li *et al*., 2018). PCA has also been used to explore ABL variables, such as boundary‐layer turbulence (Wilson, 1996; Lin *et al*., 2008), urban heat island characteristics (Vicente‐Serrano *et al*., 2005; Qiao *et al*., 2018), and air quality (Henry *et al*., 1991; Chan and Mozurkewich, 2007; Fleming *et al*., 2012; Rogula‐Kozłowska *et al*., 2015; Gupta *et al*., 2018). Further in‐depth reviews of different PCA approaches in meteorology and climatology can be found in Monahan *et al*. (2009), Schmidt *et al*. (2019), Wilks (2011), and Zhang and Moore (2015). Some studies complement PCA with cluster analysis (CA) to explore the relations between modes or with other meteorological variables (Henry *et al*., 1991; Beaver and Palazoglu, 2006) and to identify and group geographical regions according to similar target variable variability (Neal and Phillips, 2009; Smith and Phillips, 2013; Dogruparmak *et al*., 2014). Identifying regions with co‐varying meteorology or aerosol distribution based on PCA and CA can be exploited for network design by informing optimised placement of instrumentation to capture unique phenomena and maximise network utility.

In this study, our objectives are to: (a) identify the main spatial patterns of forward modelled attenuated backscatter coefficient *β*_m_ variability across a metropolitan area (London, UK), using NWP forecasts and PCA, (b) develop a method using PCA and CA to identify areas of similar *β*_m_ and inform ALC network design, and (c) understand the impact of horizontal resolution through the use of two NWP models.

## 2 METHODS

### 2.1 NWP data

The two models used are (a) the operational, convection‐permitting UK regional model (UKV, 1.5 km; Tang *et al*., 2013), and (b) the experimental London Model (LM, 300 m; Boutle *et al*., 2016). These have been applied and evaluated for London, UK (Boutle *et al*., 2016; Lapworth and Osborne, 2016; Warren *et al*., 2018). Both are specific configurations of the Met Office Unified Model (Davies *et al*., 2005). The urban surface energy balance scheme within the research LM at the time of this study was the JULES/Best one‐tile scheme (Best, 1998; Best *et al*., 2006; 2011) and within the UKV the JULES/MORUSES two‐tile scheme (Porson *et al*., 2010; Best *et al*., 2011; Bohnenstengel *et al*., 2011). The latter requires more detailed surface information and differentiates between urban canyons and roofs in a tiled scheme to better represent surface fluxes (Hertwig *et al*., 2020). The aerosol emission ancillary is derived from the 1 km native resolution National Atmospheric Emissions Inventory dataset (Neal, 2019) to produce a dry mass mixing ratio of aerosol. The emission ancillary is based on a monthly climatology that is smoothly interpolated to a day scale using a running mean. Each day, a sinusoidal function is used to vary emissions across the day, which peak at 1200 UTC. The orography ancillary is from a digital terrain elevation data model with a native 100 m resolution (Boutle *et al*., 2016). The nine land‐use tiles are populated from the Institute of Terrestrial Ecology 25 m resolution dataset (Bunce *et al*., 1990). All ancillaries are rescaled to the respective NWP model resolution for implementation.

**FIGURE 1:**
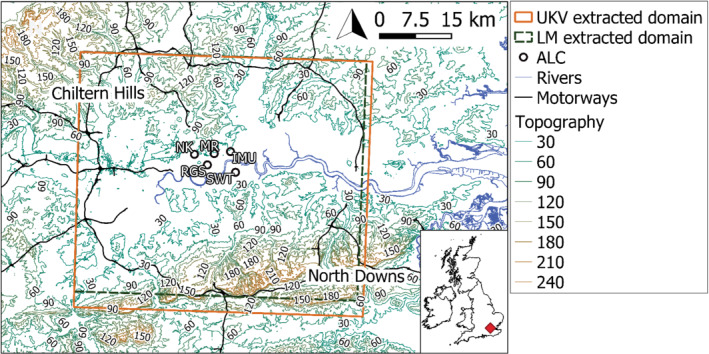
London Urban Meteorological Observatory automatic lidar and ceilometer (ALC) sites (circles with labels) within the “London” grid domains of the UKV (1.5 km) and London Model (LM; 300 m) extracted for the principal component analysis. Chiltern Hills and North Downs are highlighted as areas with significant orography. Contains Ordnance Survey data ©Crown copyright and database right (2019). Topography in metres above sea level [Colour figure can be viewed at wileyonlinelibrary.com]

In this study, 28 clear‐sky days are selected between April 1, 2018 and October 31, 2018 to avoid cloud‐related processes (Table S1). Data from both models are used to force the aerosol lidar FO (aerFO; Warren *et al*., 2018) to create three‐dimensional hourly fields of attenuated backscatter *β*_m_. Hourly aerFO calculations are conducted from midnight for 24 hr using the prior‐day 2100 UTC forecast (i.e., 3 hr spin‐up at the start of every model run are not used). The NWP data from both models are extracted for the London domain (Figure 1).

To account for diurnal variations in meteorological processes, such as the ABL height response to the surface energy balance, *day* (sunrise (SR) + 2 hr to sunset (SS) − 2 hr) and *night* (i.e., SS + 2 hr to SR – 2 hr) are analysed separately. From both NWP models, the 24 model levels from the bottom to 2,075 m above ground level (agl) are selected so as to include the ABL and (typically) the majority of the aerosol in the total atmospheric column (Seinfeld and Pandis, 2016). This ensures both the daytime boundary layer and night‐time residual layer are always within the domain analysed. The heights of the model levels represent the midpoint of each layer. In London, the median boundary layer depth (1,704 m) and the typical daytime maximum mixed‐layer height are below 2,075 m, as estimated from ALC observations within the London Urban Meteorological Observatory (Figure 1) (Kotthaus and Grimmond, 2018b). Manual quality control further ensures the ABL height does not exceed 2,075 m for any of the case‐study days selected.

### 2.2 Overview of the aerFO

Warren *et al*.'s (2020) aerFO version 2 is used to calculate *β*_m_ (at 905 nm). Briefly, the two critical variables are obtained from the NWP output: dry mass of total aerosol (*m*, kg·kg^−1^) and relative humidity (*RH*). Model fields of air temperature *T*_air_ and pressure *p*_air_ are used with the specific water vapour mixing ratio *q* to compute the water vapour absorption. Both the UKV and LM provide *m* from the passive aerosol tracer (*m*_MURK_) of the MURK visibility scheme, which considers the most common UK aerosol species (Neal, 2019). A constant lidar ratio of 43.1 sr is used. This lidar ratio, calculated specifically for urban areas, uses both in‐situ urban aerosol observations from an urban background site (North Kensington) in the London Air Quality Network (DEFRA, 2018) and modelling (Warren *et al*., 2020).

First, aerFO estimates physical properties of the aerosol in accumulation mode from *m*_MURK_, including the dry mean volume radius and total number concentration. This follows the MURK empirical parametrisation based on the climatological mean mass of dry aerosol, mean dry volume radius, and total number concentration (Clark *et al*., 2008). The climatological (monthly) values and the geometric distribution for the accumulation mode are calculated from aerosol observations at an urban background site in London (NK; Figure 1; DEFRA, 2018).

Second, aerosol optical properties are calculated, including the extinction efficiencies for the dry aerosol particles and the effect of hygroscopic growth on particle extinction. For computational efficiency, precalculated look‐up tables of dry extinction efficiency and hygroscopic growth effects are used. The estimation of the extinction coefficient includes the effect of water vapour. Third, the backscatter is calculated using a fixed lidar ratio and then converted into *β*_m_ by applying the transmission factor.

There are several sources of uncertainty in the spatial variation in *β*_m_ due to the NWP model aerosol composition being spatially invariant (in this application) that need to be considered. The lidar ratio, dry extinction efficiency, and extinction enhancement factor could vary spatially in reality, most notably when wind speeds are low and aerosol mixtures become more greatly influenced by local sources. Consequently, some spatial variability may be missed. However, the lidar ratio and extinction enhancement factor are highly sensitive to *RH*, and all three variables are highly sensitive to *m*_MURK_ (Warren *et al*., 2020), which are spatially variable, and should still allow many spatial patterns of *β*_m_ variability to be identifiable. For example, areas experiencing sea breezes also experience changes in *RH*, as well as aerosol composition, and the spatial variability in *β*_m_ may, therefore, be partially captured. To reduce the uncertainty in spatially varying aerosol proportions, speciated aerosol ancillaries can be used. However, speciated aerosol emission ancillaries were not available for the NWP models used in this study.

### 2.3 S‐mode PCA

To explore the nature of *β*_m_ variability in the LM domain (Figure 1), PCA is used to extract the most important spatial patterns from the original dataset and to identify when these spatial patterns are most prominent during the study period. As the distribution of *β*_m_ is positively skewed, it is not directly appropriate for PCA (Neal and Phillips, 2009). Therefore, a logarithmic transformation (log_10_(*β*_m_)) is applied to reduce the skewness. For example, taking the logarithmic transformation of daytime *β*_m_ at 111.7 m agl reduced the skewness from 9.85 to 0.89.

Following Wilks (2011), S‐mode PCA of log_10_(*β*_m_) is carried out for each model level separately. To begin, a vector is created containing the time series of log_10_(*β*_m_) from each grid cell for a model level (*x* = *x*_1_, …, *x*_*n*_, where *n* is the number of grid cells on the model level). *x* is then mean‐centred elementwise (*x*′). PCA of *x*′ will then identify positive and negative spatial patterns of variability with respect to the time average. The covariance matrix *S* of *x*′ is then calculated, allowing PCA to emphasise identifying the largest covariances in *x*′ as the main spatial patterns. Singular‐value decomposition can then be carried out on *S* to produce unit‐scaled eigenvectors (*e*_*i*_, where ||*e*_*i*_|| = 1) with paired eigenvalues *λ*_*i*_, where *i* = 1, …, *n*, and where *n* is the number of original spatial variables *x*. The eigenvectors are then used to calculate a set of new, uncorrelated variables (the principal components [PCs]), that relate each *e*_*i*_ to each *x*_*i*_ (length of *e*_*i*_ is equal to the number of spatial points in *x*). A PC time series can be interpreted as a series of “scores”. High positive PC scores equate to the spatial pattern *e*_*i*_ being more relevant at a given time (length of a PC time series is equal to the length of the *x* time series), whereas more negative scores indicate the inverse of *e*_*i*_ is more relevant. Thus, each successive PC explains the maximum remaining variability in the original dataset.

**FIGURE 2:**
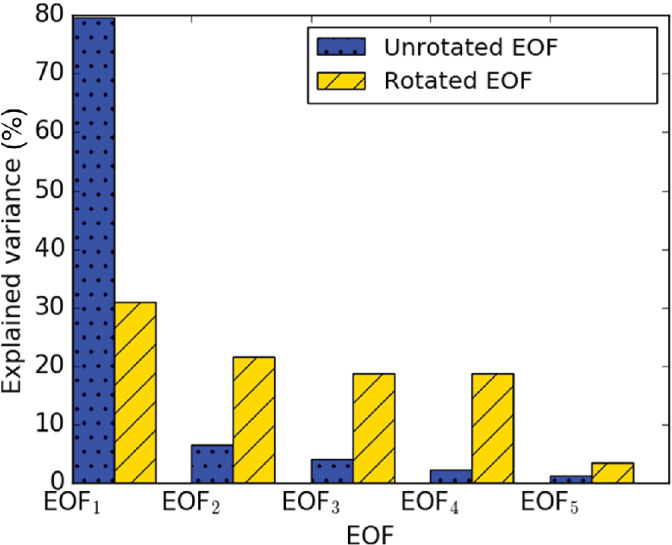
Explained variance of the first five unrotated empirical orthogonal functions (EOFs) (blue with dot hatching) and varimax rotated EOFs (red with line hatching), derived from UKV daytime forward‐modelled attenuated backscatter coefficient *β*_m_ data at 111.7 m above ground level [Colour figure can be viewed at wileyonlinelibrary.com]

By design, S‐mode PCA produces orthogonal eigenvectors that represent statistical patterns of spatial variability. The orthogonality constraint of the method means that the first eigenvector captures the maximum variability in the data, and subsequent eigenvectors sequentially partition the remaining variability (Figure 2). However, these patterns may not be readily *physically* interpretable, because statistical patterns are not guaranteed to be matched to any single physical process. In fact, a single eigenvector could contain information related to multiple physical processes. Therefore, to ease physical interpretation, varimax rotation (Kaiser, 1958; Richman, 1986; Jolliffe and Cadima, 2016) is performed on a limited number of leading vectors in *e*. Varimax finds a new eigenvector rotation that maximises the sum of the variances of the squared loadings. Effectively, varimax redistributes the explained variability and identifies a new set of eigenvectors *e*_*i*_, so that more unique spatial patterns are represented across fewer (or a single) eigenvector (Richman, 1986; Neal and Phillips, 2009; Figure 2). Here, only the *e*_*i*_ that explain more than 1% of the total variability in the original dataset are analysed. This threshold balances the need to retain as much of the original variability as possible while limiting the number of *e*_*i*_ that require physical interpretation. The selection criterion also limits the creation of multiplets (i.e., sets of *e*_*i*_ that effectively describe the same phenomena) (Wilks, 2011).

Before varimax rotation is performed, the *e*_*i*_ are scaled by $\lambda_{i}^{1/2}$ $\left(\|e_{i}\|=\lambda_{i}^{1/2}\right)$ to “load” the vectors. The individual elements of scaled eigenvectors (*e*_*i*,*k*_, where *k* = 1, …, *n*) are hereafter referred to as loadings. This scaling effectively incorporates the information about the explained variability of *e*_*i*_ into the newly rotated eigenvectors (see table 12.3 in Wilks (2011)). Although it is possible for varimax rotation to maintain the orthogonality of *e*_*i*_, the scaling used here means *e*_*i*_ are not orthogonal and may be correlated (Wilks, 2011). As the eigenvectors are created with respect to space—and to be consistent with the literature (e.g., Jolliffe and Cadima, 2016; Qiao *et al*., 2018)—the retained, rotated, and loaded eigenvectors are hereafter referred to as EOFs.

Following varimax rotation, rotated PCs are calculated using regression (Field, 2009):

(1) $${\mathrm{PC}}_{i}=x^{\prime }\left(S^{-1}\cdot {\mathrm{EOF}}_{i}\right)$$

where *x*′ is the original, mean‐centred dataset and *S* ^−1^ *⋅*EOF_*i*_ act as PC coefficients for PC_*i*_. As *S* is often an ill‐conditioned matrix and unsuitable for inversion, to calculate *S* ^−1^ we take the Moore–Penrose pseudo‐inverse of *S* using singular‐value decomposition (Strang, 1988):

(2) $$S^{-1}=V\sum^{-1}U^{T}$$

Spearman correlation coefficients *r* are calculated between EOFs and PCs to identify EOF multiplets for interpretation.

To aid interpretation, the relations between EOFs and different meteorological variables are explored. For each EOF, *x* is subsampled twice in time: when the paired PC scores are (a) above the 90th percentile and (b) below the 10th percentile. These two data distributions are assumed to represent the meteorological conditions associated with an EOF or its inverse, respectively.

### 2.4 Ward CA

CA is used with PCA output to find geographic regions of similar *β*_m_ variability, based on the spatial patterns captured. The geographic clusters that CA produces can then be interpreted to aid ALC network design. Agglomerative Ward minimum variance CA is chosen here, as this clustering method merges grid cells into groups based on their similarity (Ward, 1963; Wilks, 2011). Across the 24 model levels analysed, all the unrotated eigenvectors (uEOFs) that explain more than 1% of the total variability of their respective model level are selected and used collectively as CA inputs (i.e., 2,075 m model level and below). This will produce a single horizontal CA map. Although rotated EOFs are analysed to identify individual spatial patterns of variability (Section 3.1), uEOFs are used as input to the clustering as they contain the same statistical information on spatial variability but are statistically independent, and therefore better suited for statistical analysis.

For each *k* grid box, the unrotated eigenvector loadings from each uEOF (*e*_*i*,*k*_) are combined into new vectors. CA is performed on these new vectors. The initial *k* separate groups (each with one variable member) are iteratively paired and merged to reduce the number of clusters until *j* specified clusters remain. The clusters are merged according to the minimum sum of squared distances between all cluster variable elements and cluster centroids (i.e., merging the two most similar clusters). The loadings represent the amount of original variability explained across *x*, both positive and negative, and are larger for uEOFs that explain more. The varied loading of uEOFs also provides a benefit to the CA. Vectors scaled with larger loadings have a larger range of values, which effectively gives those uEOFs larger weights in the CA (Kaufman and Rousseeuw, 2005). This means that cluster groups are weighted more towards important uEOFs that explained more of the original variability.

Central to the network design is the number of clusters to produce by the CA. This number should be set equal to the number of sensors to be deployed in the network. As Ward CA minimises the total variance within groups (Ward, 1963), one instrument per cluster is expected to optimise representation. In our analysis, EOFs are scaled relative to the total variability at each height (i.e., not weighted with respect to other heights). Therefore, all heights are considered equally important when clustered. Alternatively, elbow, silhouette, or gap statistic methods could be used with a dendrogram to help inform the optimum number of clusters to use if the number of sensors to be deployed is not already known.

## 3 RESULTS

### 3.1 Spatial variability of forward‐modelled attenuated backscatter *β*_m_

**FIGURE 3:**
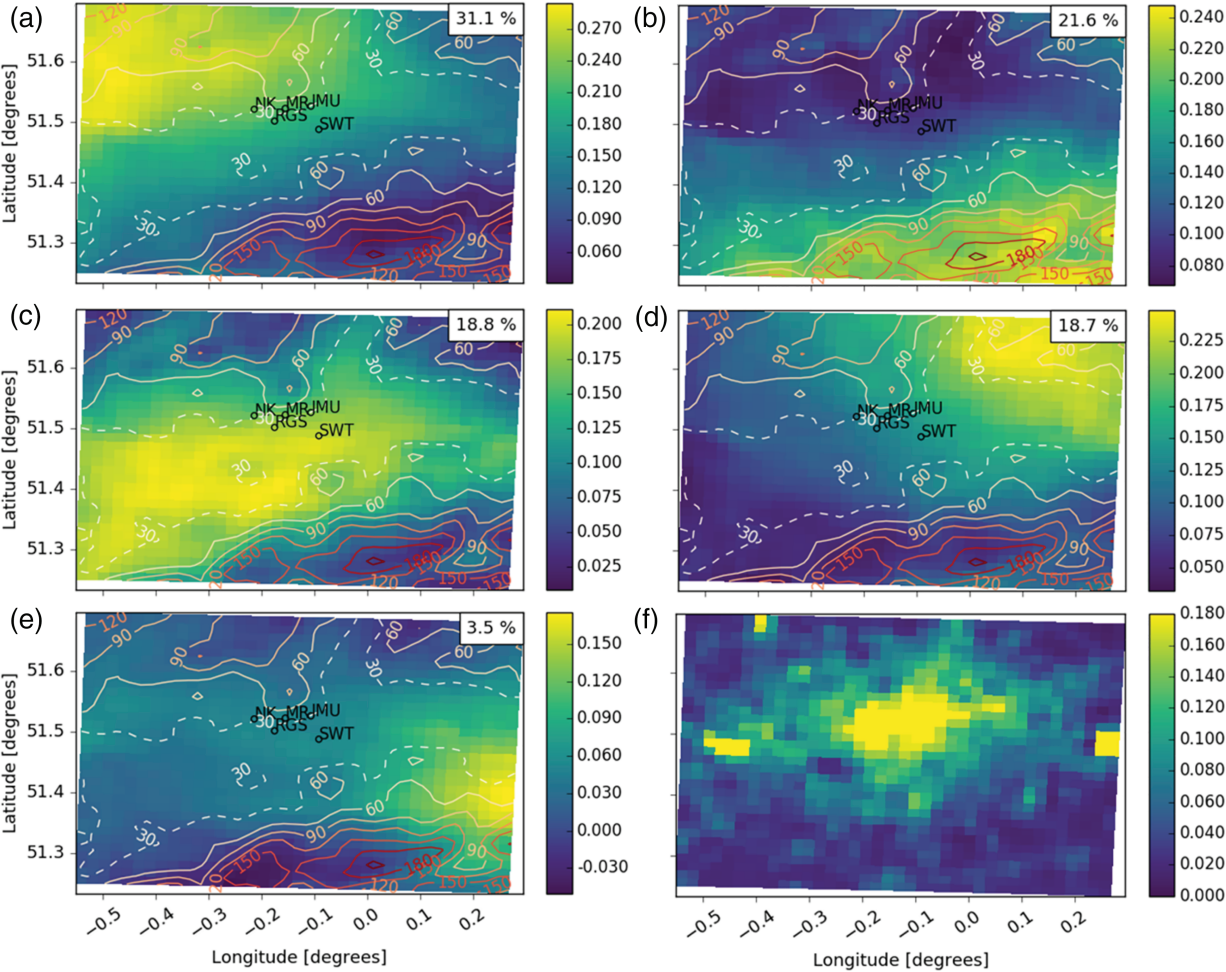
Examples of the main loaded, rotated empirical orthogonal function (EOF) patterns that typically occur across different model heights, derived from the UKV (1.5 km) during the daytime (colour) with topographic contours (lines, 30 m dashed). EOFs explain decreasing variability (% in top right) of the original dataset (111.7 m above ground level): (a) EOF_1_; (b) EOF_2_; (c) EOF_3_; (d) EOF_4_; (e) EOF_5_; (f) *m*_MURK_ background aerosol emissions (μg·m^−2^·s^−1^) climatology for July. London Urban Meteorological Observatory automatic lidar and ceilometer network (Figure 1) shown as dots and labels in the domain centre

**FIGURE 4:**
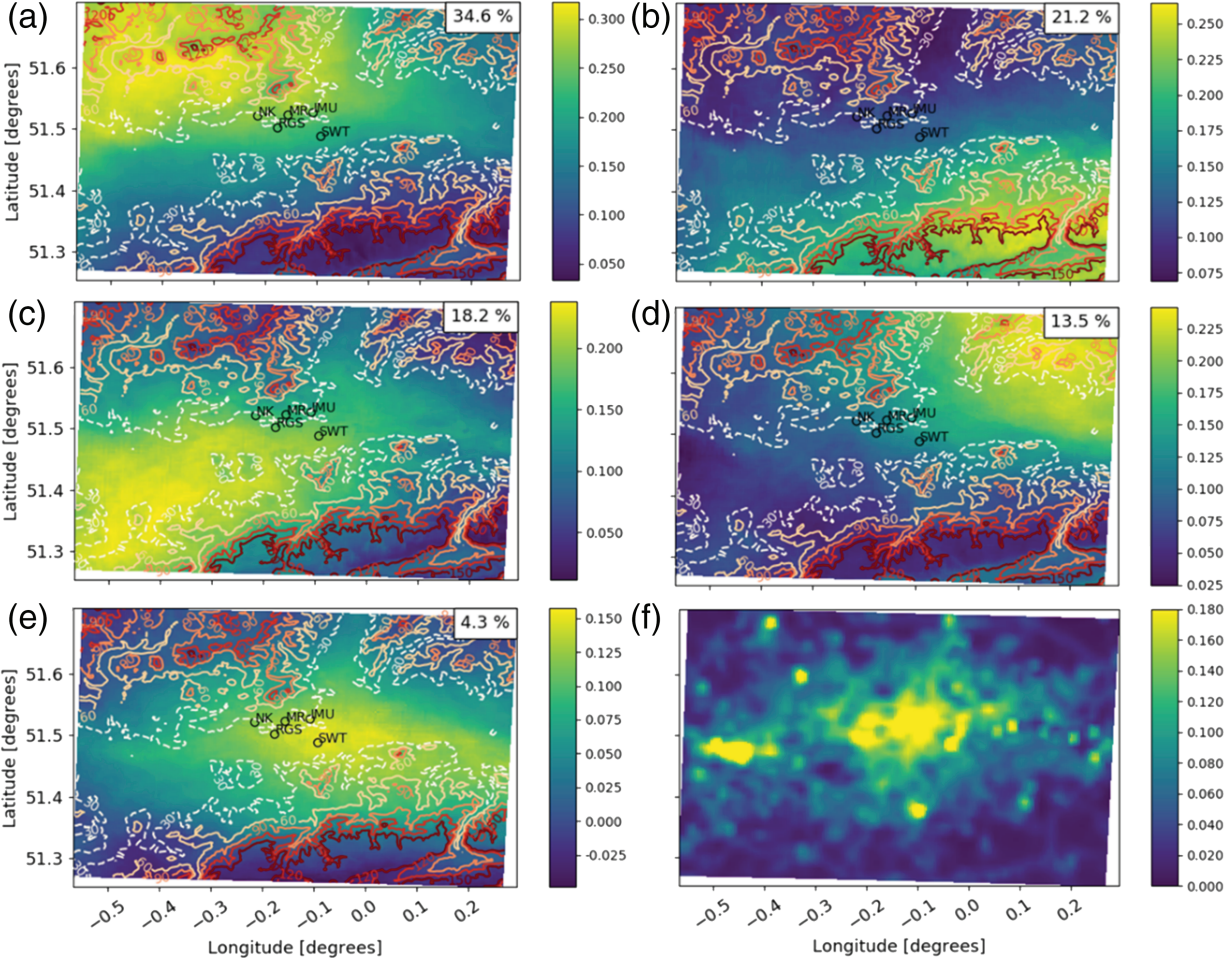
As Figure 3, but derived from the London Model (300 m)

The PCA with varimax rotation for the daytime period produced between four and seven EOFs for each model level in both models that explained more than 1% of variability in the original dataset. In general, similar EOF patterns are identified across different heights. Examples of common daytime EOF patterns from the UKV (Figure 3) and the LM (Figure 4) are shown for the 111.7 m agl model level. At this height, five EOFs are needed to explain the *β*_m_ variance.

**FIGURE 5:**
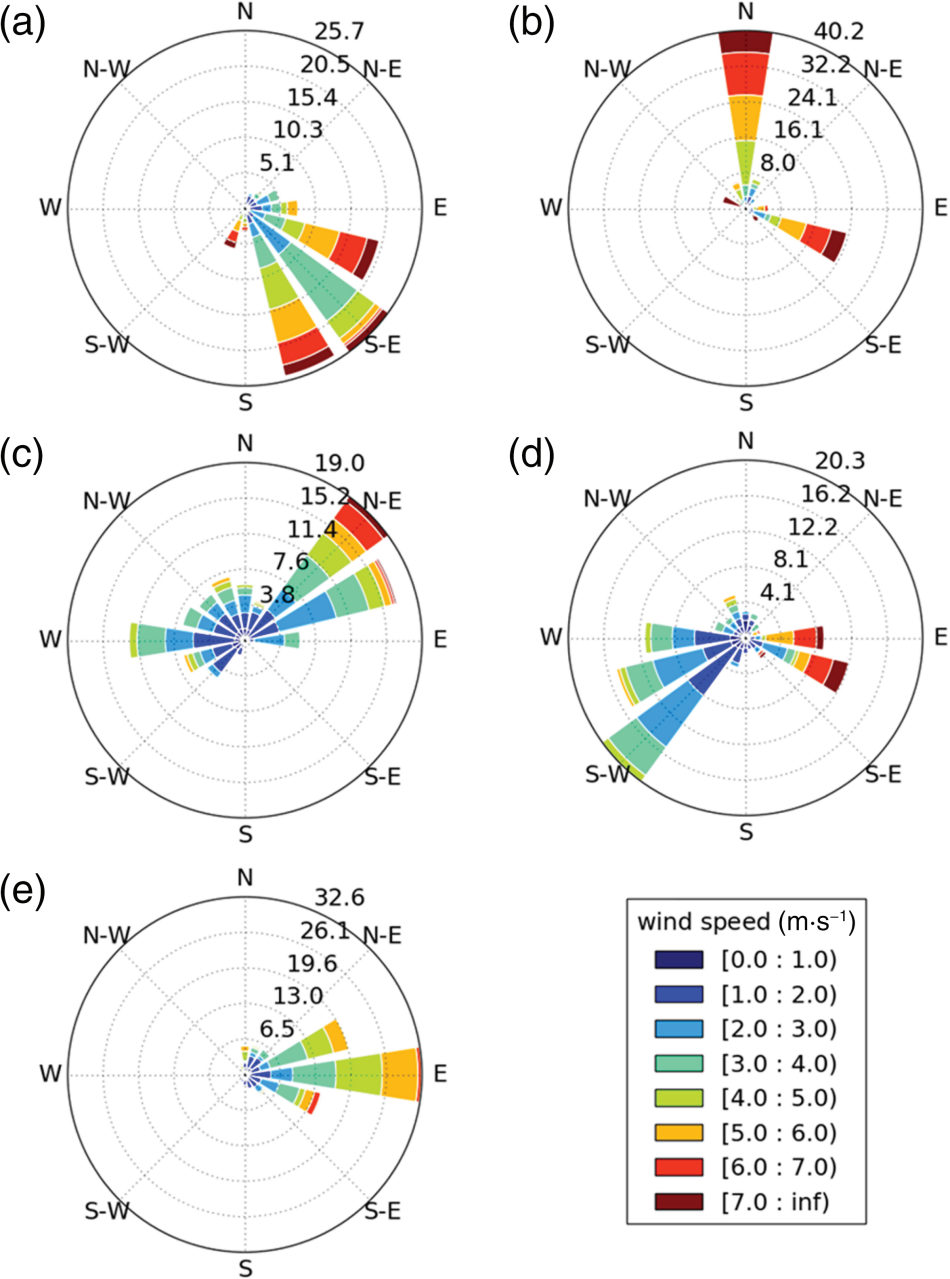
Paired daytime wind roses of UKV model wind speed (m·s^−1^) and direction (degrees) for each rotated empirical orthogonal function (EOF) at model height 111.7 m (Figure 3): (a) EOF_1_; (b) EOF_2_; (c) EOF_3_; (d) EOF_4_; (e) EOF_5_. Wind speeds are temporally sampled when the scores for each PC are above the 10th percentile, and spatially sampled across the domain (wind characteristics most associated with each EOF). Bins extend from low inclusive and high exclusive (key). Radial axis (%) frequency of occurrence by wind direction bin. See Figure S1 for a composite of all wind speeds

EOF patterns derived from both NWP models are strongly related to wind speed and direction. The highest loadings in each EOF (Figures 3a–e and 4a–e) are associated with areas downwind of aerosol emission sources (cf. Figures 3f and 4f) leading to an increase in aerosol and, consequently, *β*_m_. As most aerosol emission sources are located near the centre of the domain (i.e., the most urbanised area), the highest EOF loadings are usually found near the domain edges. For example, the highest loadings in EOF_1_ are in the northwest of the domain (Figure 3a) as EOF_1_ is most prevalent under south–southeasterly winds (Figure 5a). The 28 case‐study days used to derive the EOFs cover a wide range of wind directions and speeds (Figure 5), and therefore the importance of horizontal advection to *β*_m_ variability is highly likely at these NWP scales. A full day and night composite of wind speed and direction over all cases is in Figure S1.

A second factor determining the shape of the EOFs is topography. London, located in a river valley, is situated between the Chiltern Hills (north) and North Downs (south and southeast) (Figure 1), so its elevation is relatively low compared with its surroundings (>200 m above sea level; Figures 3a–e and 4a–e). The EOF_1_, EOF_2_, and EOF_4_ spatial patterns occur when air is advected onto the hills in the northwest, southeast, and northeast, respectively. As the air is forced upward, part of the detected *β*_m_ variability is explained by the *RH* response to air temperature reduction. But the advected air also increases *m*_MURK_ locally, where background emissions are typically low. As exp(*β*_m_) is proportional to *RH* and *β*_m_ ∝ *m*_MURK_, the advection increases the local variation of *β*_m_. For example, if an air parcel with *m*_MURK_ = 24 μg·kg^−1^ is advected onto a hill with background concentration of *m*_MURK_ = 18 μg·kg^−1^, and raised adiabatically by 100 m such that *RH* increases from ∼80 to 85%, *β*_m_ increases locally by ∼1.2 × 10^−6^ m^−1^·sr^−1^. This combined orographic effect and advection of aerosol emitted from the major sources (middle of the domain; Figure 3f) increases *β*_m_ in areas surrounding the city. The spatial variability of *β*_m_, *m*_MURK_, and *RH* for EOF_1_ from the UKV is shown in Figure S2.

The general distribution of loadings in the EOFs across the London domain are similar for the two NWP models, as the synoptic winds are the most important factor driving the spatial patterns. Whereas the LM EOFs have more spatial detail in the loading distribution around complex orography (Figure 3b cf. Figure 4b), the UKV topographic resolution is sufficient to generate orographic uplift and the associated peak in loadings.

EOF_1_ and EOF_2_ for both NWP models, across each of the model levels analysed, explain a large amount of the total variability (typically between ∼25–40% for EOF_1_ and ∼20–40% for EOF_2_). EOF_3_ explains ∼13–25% of the total variability in *β*_m_ and can be linked to two processes that increase *β*_m_: relatively higher *RH* in the southwest, and high aerosol variability in the centre of the domain. This EOF often occurs with low winds speeds or northeasterlies (Figure 5). In the UKV above 471.7 m, the two processes are instead represented by two separate EOF patterns, here labelled as EOF_3_ sub‐patterns, EOF_UKV,3–1_ (peak in loadings in the southwest of the domain driven by *RH*) and EOF_UKV,3–2_ (peak loadings in the centre of the domain driven by urban aerosol exchange) (Figure S4). In the LM, EOF_LM,5_. is most similar to EOF_UKV,3–2_ from the UKV, with respect to the loading spatial distribution.

EOF_UKV,3–2_ and EOF_LM,5_ likely portray rural–urban differences in vertical aerosol transport and changes in *RH*. PC time series corresponding to these EOFs peak with lower wind speeds, suggesting vertical aerosol transport is more important when wind speeds are low. Positive PC scores indicate higher *β*_m_ over the city, and negative scores indicate higher *β*_m_ in the surrounding rural areas. Below 111.7 m (not shown), soon after SR, the PC scores are high, which reflects the greater build‐up of *m*_MURK_ overnight in urban areas compared with the rural surroundings. Greater buoyancy over built‐up areas due to greater urban heat and roughness, in the absence of higher wind speeds, encourages greater upward transport of *m*_MURK_ and *β*_m_ in the domain centre. This vertical redistribution reduces the near‐surface build‐up of *m*_MURK_, and consequently emphasises the urban–rural difference.

Above 111.7 m, EOF_UKV,3–2_ and EOF_LM,5_ PC scores typically have a diurnal pattern, and are low after SR, peak midday, and decrease before SS (Figure S5a). Furthermore, the scores also increase later after SR and decrease sooner before SS for level heights further from the surface (Figure S5b). This smaller range (later rise, lower fall) of PC scores at greater heights could reflect the extra time required for boundary‐layer mixing processes to reach greater heights and to entrain the existing residual layer while redistributing relatively high near‐surface concentrations of *m*_MURK_ further vertically. Again, this suggests the importance of *m*_MURK_ emissions and ABL dynamics in determining the variability of *β*_m_.

EOF_LM,5_ tends to represent a greater proportion of total *β*_m_ variability than its UKV counterpart EOF_UKV,3–2_, as urban effects are better resolved by the higher resolution of both the urban characteristics and aerosol emission sources in the LM ancillaries. Aerosol and emission ancillaries with a native resolution of 1 km are coarsened for use in the UKV (to 1.5 km) and interpolated for the LM (to 300 m). Higher resolution LM ancillaries are likely to provide greater variability in *β*_m_, particularly where emission sources are more heterogeneous.

For the nocturnal analysis, as with the daytime, the EOFs are derived from PCA with varimax rotation. Again, EOFs relate strongly to wind speed, wind direction, emission source location, and orography (not shown), with peak loadings in similar positions. However, there are some differences between the daytime and night‐time EOFs.

**FIGURE 6:**
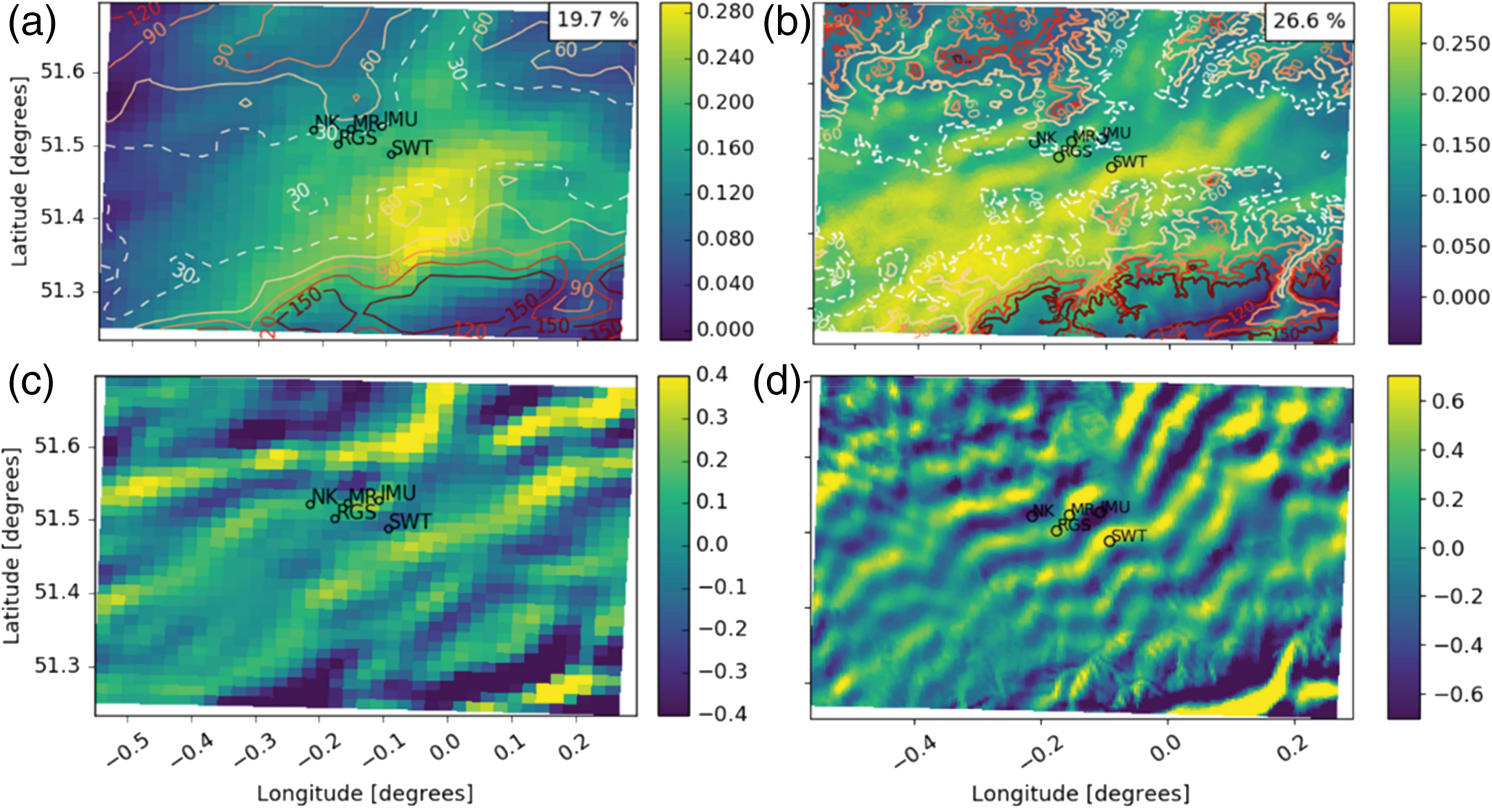
Nocturnal empirical orthogonal function EOF_2_ at the 471.7 m model level derived separately from (a) UKV and (b) London Model. Horizontal cross‐section of *w*‐wind component when EOF_2_ principal component scores are >4 on October 23, 2018, 2300 UTC (at 471 m) from (c) UKV and (d) London Model

Between model heights 325.0 and 955.0 m, several EOFs display wavelike patterns that are not present during the day; for example, EOF_2_ at 417.7 m in both models (Figure 6), which generally occurs under northwesterly winds. Similar wave structures are found in EOFs associated with southerly winds (not shown). These patterns likely represent gravity waves produced as northwesterly flow passes over the Chiltern Hills (northwest London domain; Figure 3f) (Figure 6a), which is in agreement with earlier studies using UKV and LM data for the Greater London area (Lapworth and Osborne, 2016; 2019). Gravity waves can occur in statically stable conditions and have been found in EOFs derived from large‐eddy simulations of the planetary boundary layer under weakly convective conditions (Wilson, 1996). Using lidar observations Gibert *et al*. (2011) found gravity waves to cause fluctuations in *RH*, which would translate into *β*_m_ variability captured by the EOFs.

The presence of waves is visible in the *w*‐wind component when the PC scores for EOF_2_ are >4 (e.g., Figure 6c,d). Under clear‐sky conditions at night, the boundary layer is more likely to be stable, which can reinforce the presence of gravity waves (Wallace and Hobbs, 2006). The unstable daytime conditions limit their formation (Figures 3 and 4). However, the frequency and spatial prevalence of the gravity waves in the NWP model data may be too large compared with reality, leading to an overrepresentation in the EOFs. Both models have been found to overestimate atmospheric stability over the urban area of London, which is caused by an underestimation of the anthropogenic heat emissions (Bohnenstengel *et al*., 2014). Further, the simpler one‐tile urban surface scheme used for the LM simulations also tends to underestimate London's sensible heat fluxes in the evening (Hertwig *et al*., 2020). Thus, the gravity waves are likely a less important phenomenon than implied by the EOFs derived from the NWP model data.

### 3.2 Application of spatial variability to network design

**FIGURE 7:**
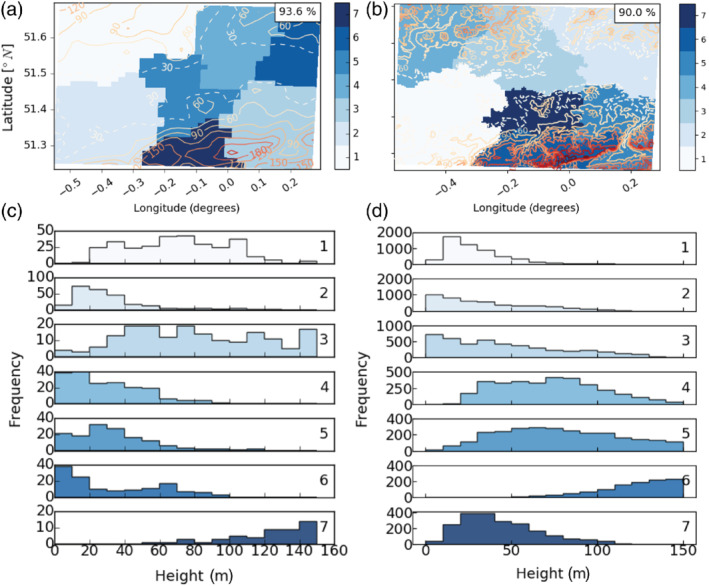
Ward cluster analysis (*n* = 7 clusters) of unrotated empirical orthogonal function (EOFs) from principal component analysis of daytime *β*_m_. Colour patches numbered from the largest (C_1_) to the smallest (C_7_) derived from (a, c) UKV and (b, d) London Model; (a, b) location of cluster with topographic contours (lines, 30 m dashed), and (c, d) topographic variation in each cluster. Note frequency (*y*‐axis) varies. All EOFs included explain ≥1% of the variability in modelled attenuated backscatter coefficient [Colour figure can be viewed at wileyonlinelibrary.com]

To find geographic regions with similar *β*_m_ variability, Ward CA (Ward, 1963; Wilks, 2011) is performed using the uEOFs for day/night, and for UKV/LM separately, with the cluster numbers (*n*) set to 5, 7 and 20. For example, Figure 7 shows the daytime UKV clustering of *n* = 7 clusters, ordered from the largest (1) to the smallest area (7).

The UKV and LM cluster maps are similar. Both have clusters located over the city centre and in the surroundings for different wind directions (Figure 7). The clusters are spatially confined, which is likely because the covariance of eigenvector values between neighbouring grid cells is relatively high compared with grid cells that are further apart. The central LM cluster (7, C_LM_,_7_) is smaller than its UKV counterpart (C_UKV,5_), which could be related to the higher loadings over central London compared with its surroundings in the first LM uEOF, and more nuanced skill possible with the smaller grid resolution. The clusters split into three elevation range types (Figure 7c,d): (a) wide range, in the northwest and southeast; (b) small range and low elevation, for most of the others; and (c) predominantly higher elevation, in the south.

Qualitatively, the boundaries between adjacent clusters follow the topographic contours for *n* = 7 (notably C_UKV,1_, C_UKV,7_ and C_LM,5_, C_LM,6_; Figure 7), which is the same for other cases (*n* = 5 or *n* = 20) analysed. When *n* = 20 (Figure S6), the smaller clusters align with more detailed topographic features (e.g., valley variations and hill tops, C_UKV,12_ in the southwest). CA results are also similar between day and night, despite the wavelike patterns in night‐time EOFs (Figure S7).

To better understand the near‐surface *β*_m_ variability (cf. variability in the full vertical domain as used in Figure 7, additional daytime cluster maps are produced, using EOFs from model height of (a) 5.0 m only, and (b) 5.0–111.7 m inclusively (Figure S8). Unlike the clusters in Figure 7, near‐surface clusters have a greater east–west elongation and appear to be more tightly constrained by local near‐surface emission sources, which also have an east–west elongation due to the presence of Heathrow Airport (west), the city centre (middle), and several emissions sources near the River Thames (east) (Figures 4f and 5f). Comparison of near‐surface clusters with clusters in Figure 7 highlight that near‐surface *β*_m_ variability is influenced more by local‐scale emission sources, whereas clusters derived using the full sampled height domain are affected by larger‐scale processes.

The CA maps can be used to inform the deployment of ALC instruments. As Ward clustering aims to minimise the variability in *β*_m_ within a cluster, one logical approach would be to locate a single ALC instrument in each cluster. Using the maps and wind roses together to assess aerosol advection patterns can help prioritise deployment. For example, larger clusters and those with frequent upwind advection would come first. A network of seven ceilometers deployed for data assimilation into the UKV could be distributed with one in the city centre, to observe the urban plume, and the other six in the surrounding hills (one in each cluster). Instruments in rural areas would monitor both advected aerosol emissions upwind from the city and the subsequent orographic lifting effect. If only two instruments are deployed, the CA maps derived in this study would suggest one in the city (C_UKV,5_), and a second in the northwest cluster (C_UKV,1_) given the relatively large cluster size and high frequency of southeasterlies. At night, the cluster shapes and areal extents can change. Therefore, where possible, the recomendation is to use both day and night CA maps when selecting a site location.

## 4 CONCLUSIONS

To assess what drives spatial variations in aerosol (and *RH*), within the boundary layer across a region in and around a megacity under cloud‐free conditions, a method is developed to identify common spatial patterns of variability. The attenuated backscatter coefficient is modelled using the aerFO operator with input from two NWP models at different resolutions: the 1.5 km Met Office UKV and the 300 m research LM for a domain around Greater London, UK. PCA with varimax rotation is used for two periods (day, night) to create EOFs for each model level.

PCA results are combined with CA to identify areas of similar aerosol variability. The CA results can be used with wind roses to identify potential locations for ALC instruments to maximise uniqueness of observations across a network, with respect to observing features at spatial scales larger than the model resolution used, for the purpose of data assimilation and evaluation.

Common patterns in attenuated backscatter coefficient variability are identifiable across different model levels during both day and night, with similar results for both NWP models.

From the analysis of 28 days clear‐sky case‐study days, it is concluded that:

- Spatial variability in modelled attenuated backscatter coefficient *β*_m_ can be mostly explained by orography, transport of aerosols from source locations (mainly in city centre) and wind direction.
- The urban–rural difference in attenuated backscatter coefficient is most pronounced under low wind speeds and advection, allowing vertical boundary‐layer dynamics to redistribute relatively higher urban near‐surface aerosol concentrations over the city.
- Possible gravity waves influence the spatial variability of attenuated backscatter coefficient in the residual layer at night in both NWP models, through fluctuations in *RH*.
- Results with the coarser resolution NWP model (1.5 km) are comparable to the higher resolution (∼300 m) NWP model, though the higher resolution NWP benefits from higher resolution orography.
- NWP models could potentially indicate more variability in the attenuated backscatter coefficient across a city if model inputs (i.e., ancillaries) have higher resolution (e.g., aerosol emissions). For example, improved urban energy balance fluxes from anthropogenic heat emissions and heat storage could improve the representation of urban–rural contrasts.
- CA identifies distinct regions (clusters) of similar attenuated backscatter coefficient variability to inform instrument placement of a network for data assimilation into NWP models.
- CA results are similar between day and night, despite the wavelike patterns in night‐time EOFs.
- When creating five or more clusters, the cluster shape and sizes relate to orography, aerosol emissions, and wind direction. One cluster is located in the domain centre (city), and others in surrounding rural areas with high orography.
- Near‐surface clusters relate more to aerosol emissions due to greater local‐scale influence, whereas clusters using the full vertical information can be influenced more by larger‐scale influences.
- We recommend placing one instrument in the city to observe the urban plume and others in the rural surroundings, with priority given to the larger clusters and clusters with higher frequency of downwind aerosol advection from the city.
- Identification of distinct regions is mostly constrained by NWP model resolution and the spatial scales of features it can effectively resolve. To inform network design for ALCs to observe smaller spatial features, higher resolution NWP models are needed.

The PCA‐CA technique is highly adaptable and could be modified or used in a wider variety of applications. The technique could be used on subsamples of NWP data to focus on better capturing information for particular meteorological situations or regions. For example, subsampling for above‐average aerosol events, or the upper extent of the boundary layer where observations can be sparse in urban areas (Barlow, 2014). In addition, the PCA‐CA technique could aid the spatial interpretation of verification statistics. The CA highlights spatial regions where the model grid cells covaried most similarly; therefore, the spatial applicability of verification statistics using ALC instruments located in identified cluster regions can be better understood. The PCA‐CA technique could be applied to other meteorological variables, beyond aerosols (e.g., cloud base and boundary‐layer heights), to inform network design that can target variability in those variables.

As two NWP models were used, the sensitivity of cluster maps to model biases was only partially explored. Therefore, future work should consider generating cluster maps from more NWP models to better understand this sensitivity and ideally reduce the impact of model bias on instrument placement.

## AUTHOR CONTRIBUTIONS

**Elliott Warren:** Conceptualization; formal analysis; investigation; methodology; software; visualization; writing – original draft; writing – review and editing. **Cristina Charlton‐Perez:** Conceptualization; investigation; supervision; writing – review and editing. **Humphrey Lean:** Conceptualization; investigation; supervision. **Simone Kotthaus:** Conceptualization; investigation; supervision. **Sue Grimmond:** Conceptualization; investigation; supervision; writing – review and editing.

## Supporting information


**Appendix S1**: Supporting Information.
