# Supplementary material for: Spatial variability of forward modelled attenuated backscatter in clear‐sky conditions over a megacity: Implications for observation network design
Source: Q J R Meteorol Soc. 2022 Mar 10;148(744):1168–83. doi: 10.1002/qj.4253 (PMC9313619; doi:10.1002/qj.4253)
Supplement: Supplementary file 1 — Appendix S1: Supporting Information. [file QJ-148-1168-s001.docx]

Supporting Information

Table S 1: Days in each month of 2018 used for the analysis. Plots of daily observed ALC attenuated backscatter coefficient were reviewed from the London Urban Meteorological Observatory archive (LUMO, <http://micromet.reading.ac.uk/>) to identify clear days absent of hydrometeors.

| April | May | June | July | August | Sept | Oct |
| --- | --- | --- | --- | --- | --- | --- |
| 06 | 05 | 22 |  | 02 | 01 | 07 |
| 18 | 06 | 23 |  | 03 | 02 | 10 |
| 19 | 07 | 24 |  | 04 | 03 | 20 |
| 20 | 14 | 25 |  | 05 |  | 23 |
|  | 15 | 26 |  | 06 |  |  |
|  | 19 |  |  |  |  |  |
|  | 20 |  |  |  |  |  |


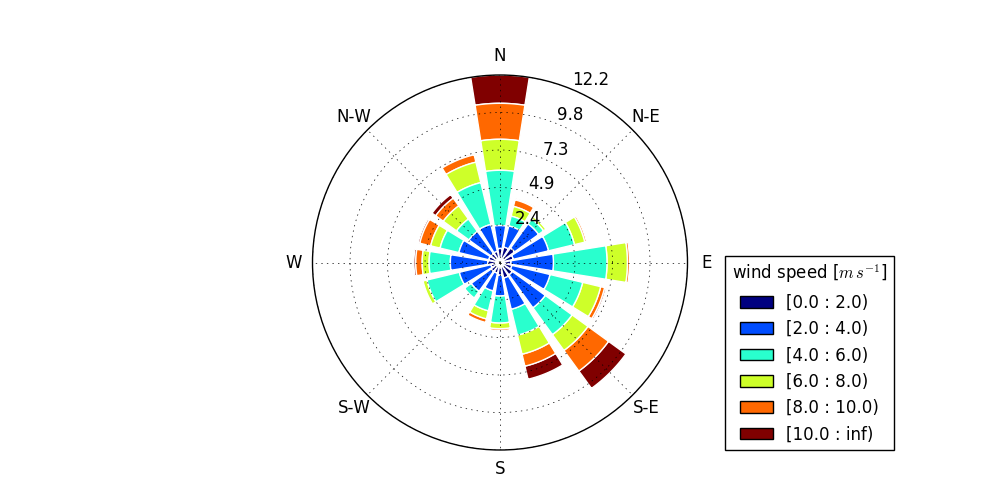


Figure S 1: Combined day and night-time wind rose (28 days) UKV model wind speed [m s^-1^] and direction (°) at model height 111.7 m across the domain. Bins extend from low inclusive and high exclusive (key). Radial axis (%) frequency of occurrence by wind direction bin. Note bin sizes differ from Figure 5.


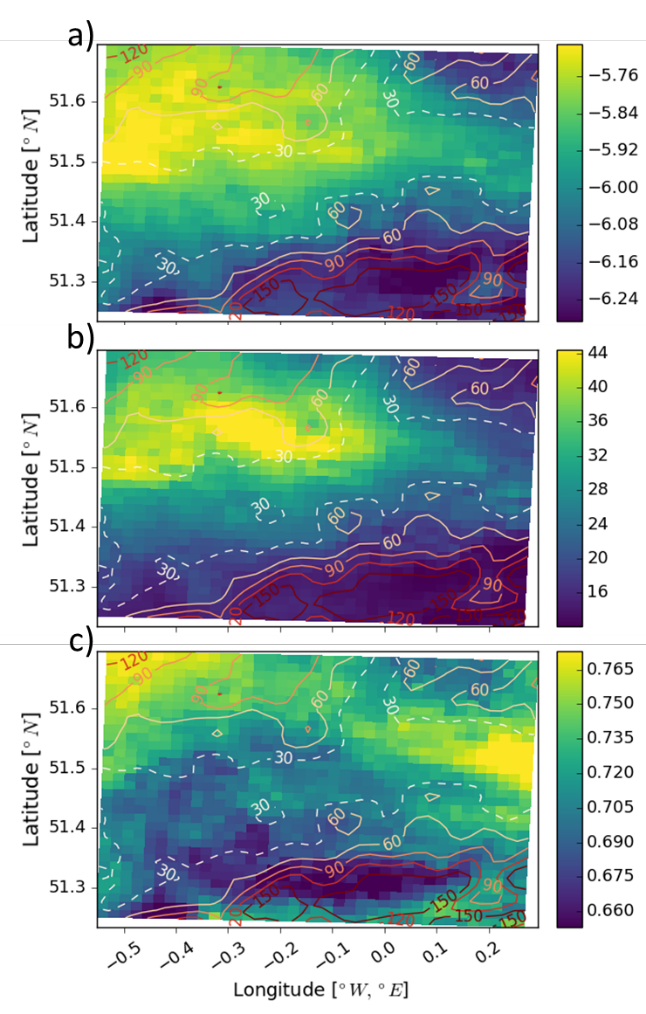


Figure S 2: Median with respect to time of (a) log_10_(βm) [m^-1^ sr^-1^], (b) m_MURK_ [μg kg^-1^], and (c) RH [fraction] at model height 111.7 m above ground level across the entire London domain in the UKV (1.5 km) during daytime, when the PC scores for EOF_1_ are above the 10^th^ percentile.


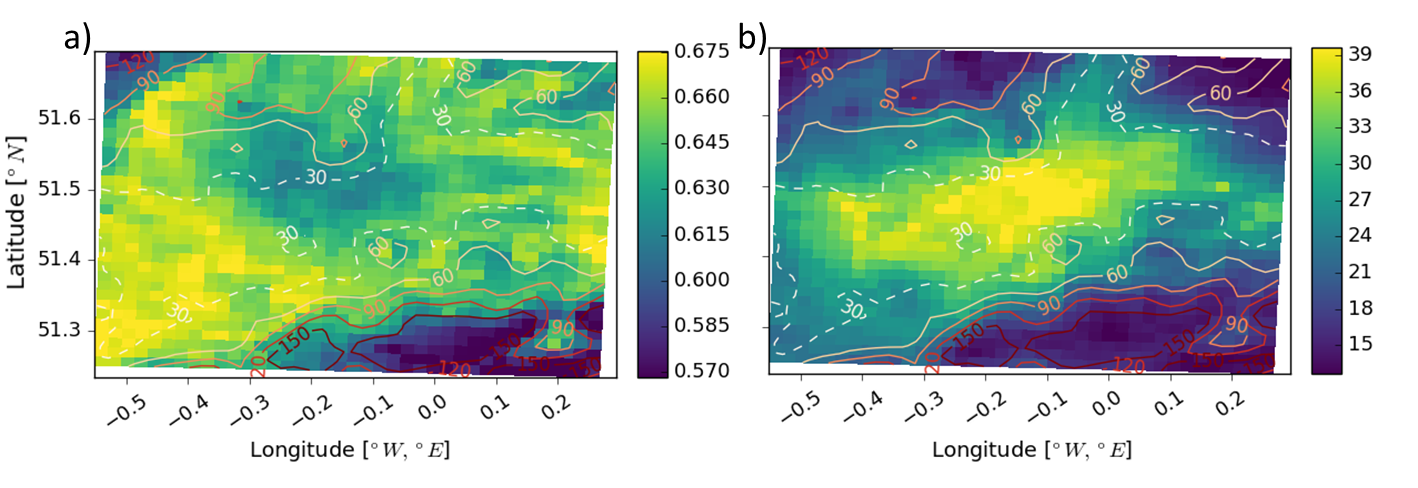


Figure S 3: Median with respect to time of (a) RH [fraction], and (b) m_MURK_ [μg kg^-1^], at model height 111.7 m above ground level across the entire London domain in the UKV (1.5 km) during daytime, when the PC scores for EOF_3_ are above the 10^th^ percentile.


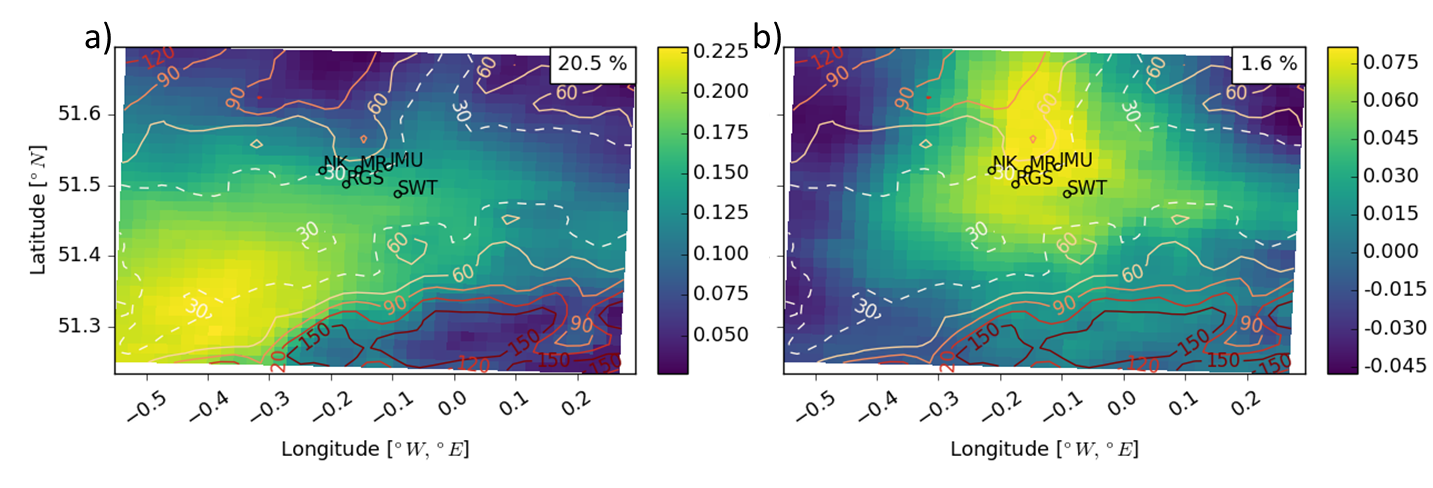


Figure S 4: (a) EOF_3-1_ and (b) EOF_3-2_ from the UKV (1.5 km) during the daytime (colour) at 471.7 m above ground level. Topographic contours (lines, 30 m dashed). EOFs explain decreasing variability (% in top right) of the original dataset. LUMO ALC network (Figure 1) shown as dots and labels in the domain centre.


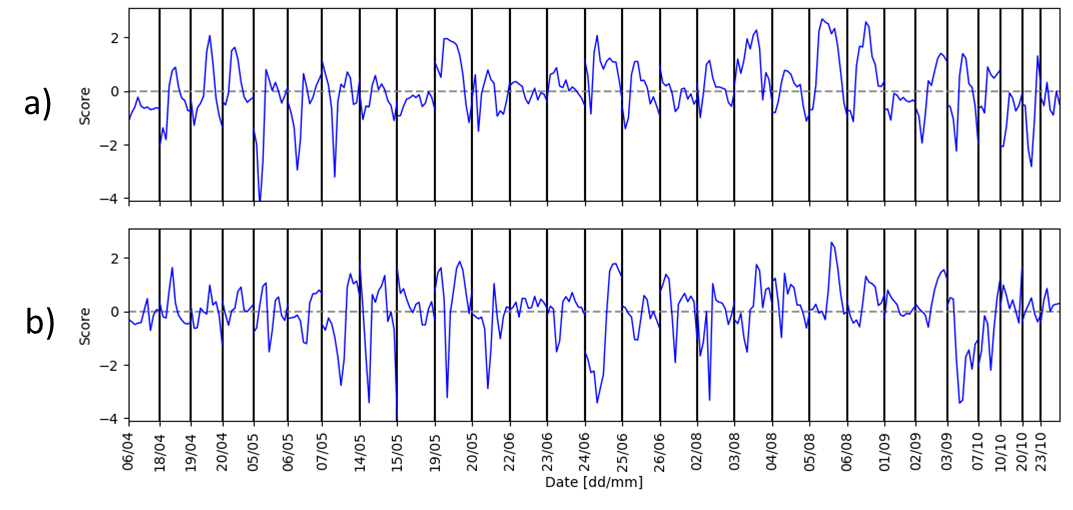


Figure S 5: Daytime hourly principal component scores for all 28 days EOF_LM.5_ (Figure 4e) for a) 261.7 m and b) 741.7 m. Note these dates are often not continuous and daylength varies with time of year.


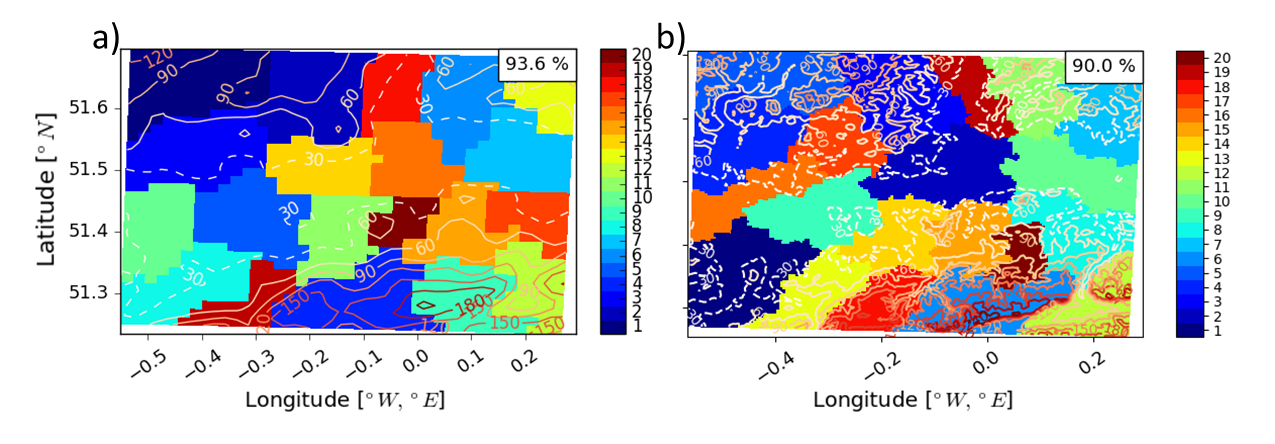


Figure S 6: Ward cluster analysis (n=20 clusters) (of unrotated EOFs from PCA) of daytime β_m_. Colour patches numbered from the largest (C_1_) to the smallest (C_20_) derived from (a) UKV and (b) LM. Topographic contours (lines, 30 m dashed). All EOFs included explain ≥ 1% of the variability in modelled attenuated backscatter coefficient.


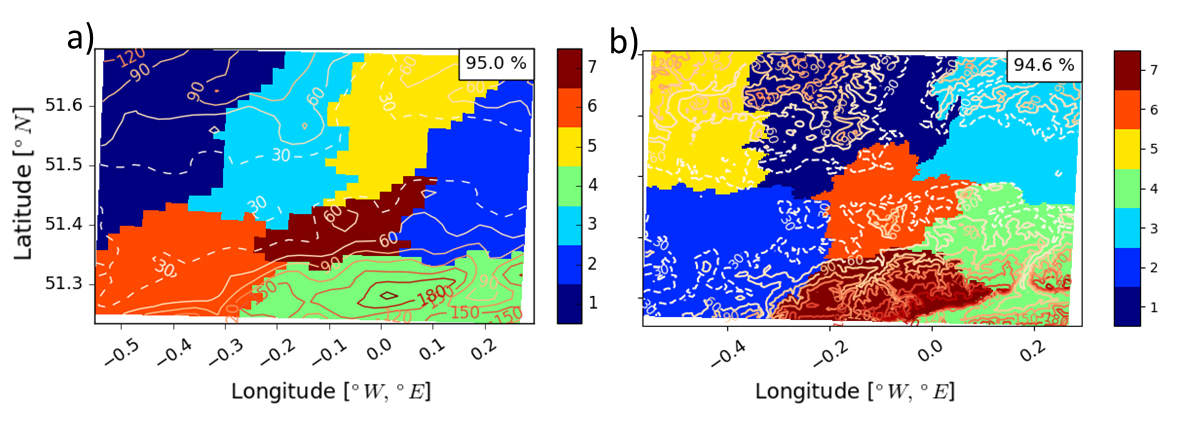


Figure S 7: Ward cluster analysis (n=7 clusters) (of unrotated EOFs from PCA) of night-time β_m_. Colour patches numbered from the largest (C_1_) to the smallest (C_7_) derived from (a) UKV and (b) LM. Topographic contours (lines, 30 m dashed). All EOFs included explain ≥ 1% of the variability in modelled attenuated backscatter coefficient.

The nocturnal CA (Figure S 7) are similar to the daytime results (Figure 7a,b) in terms of cluster shapes and sizes. Both indicate the importance of wind, relative aerosol emission location and orography on the EOFs, which do not depend highly on time of day (section 3.1). *C_UKV,7_* has a slight elongation in the south-west to north-east direction which qualitatively aligns with the relatively high MURK ancillaries in the central domain. This is likely because of the aerosol build up over time in the surface layer at night strongly affecting the lower model level uEOFs. The elongated *C_UKV,7_* shape could also be related to the nocturnal gravity wave features, but the effect would likely be limited as the *β_m_* variability due to the waves is small in the uEOFs.


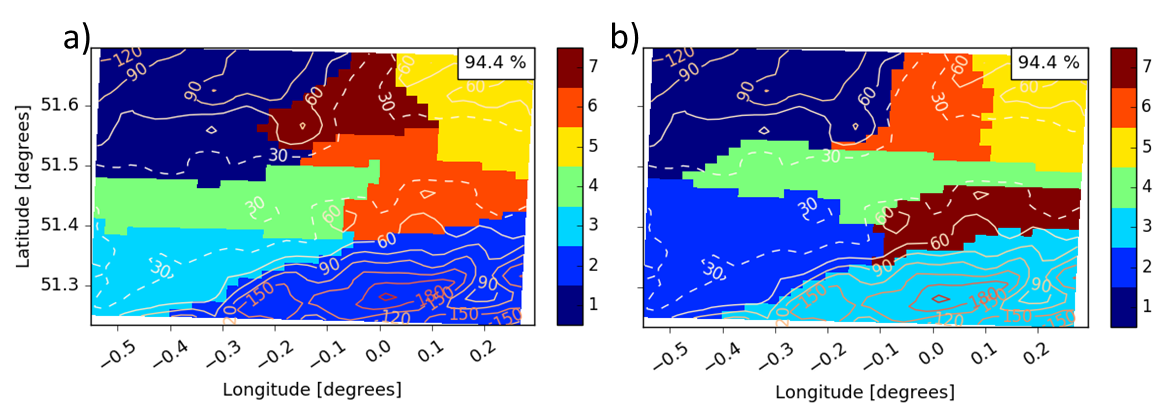


Figure S 8: Ward cluster analysis (n=7 clusters) using a sample of unrotated EOFs from PCA of daytime β_m_, derived from UKV. Clustering using EOF subsampled from model heights (a) 5.0 m only, (b) 5.0 – 111.7 m inclusively. Colour patches numbered from the largest (C_1_) to the smallest (C_7_). All EOFs included explain ≥ 1% of the variability in modelled attenuated backscatter coefficient.
